# Liraglutide Attenuates Nonalcoholic Fatty Liver Disease by Modulating Gut Microbiota in Rats Administered a High-Fat Diet

**DOI:** 10.1155/2020/2947549

**Published:** 2020-02-18

**Authors:** Ningjing Zhang, Junxian Tao, Lijun Gao, Yan Bi, Ping Li, Hongdong Wang, Dalong Zhu, Wenhuan Feng

**Affiliations:** ^1^Medical School of Southeast University Nanjing Drum Tower Hospital, Nanjing, China; ^2^Department of Endocrinology, Drum Tower Hospital Affiliated to Nanjing University Medical School, Nanjing, China

## Abstract

This study aimed to determine whether modulation of the gut microbiota structure by liraglutide helps improve nonalcoholic fatty liver disease (NAFLD) in rats on a high-fat diet (HFD). Rats were administered an HFD for 12 weeks to induce NAFLD and then administered liraglutide for 4 additional weeks. Next-generation sequencing and multivariate analysis were performed to assess structural changes in the gut microbiota. Liraglutide attenuated excessive hepatic ectopic fat deposition, maintained intestinal barrier integrity, and alleviated metabolic endotoxemia in HFD rats. Liraglutide significantly altered the overall structure of the HFD-disrupted gut microbiota and gut microbial composition in HFD rats in comparison to those on a normal diet. An abundance of 100 operational taxonomic units (OTUs) were altered upon liraglutide administration, with 78 OTUs associated with weight gain or inflammation. Twenty-three OTUs positively correlated with hepatic steatosis-related parameters were decreased upon liraglutide intervention, while 5 OTUs negatively correlated with hepatic steatosis-related parameters were increased. These results suggest that liraglutide-mediated attenuation of NAFLD partly results from structural changes in gut microbiota associated with hepatic steatosis.

## 1. Introduction

With the drastic increase in the prevalence of obesity, nonalcoholic fatty liver disease (NAFLD), characterized by excessively ectopic lipid accumulation in hepatocytes, has become the major cause of chronic liver disease in Western countries [1, 2]. NAFLD is closely associated with insulin resistance and is, therefore, generally comorbid with type 2 diabetes (T2DM). Patients with NAFLD and T2DM are more likely to experience disease progression to nonalcoholic steatohepatitis (NASH), the subsequent stage of NAFLD. Other than lifestyle-based interventions, which facilitate the loss of >5% body weight, there are no efficient treatments for NAFLD [3].

The gut microbiota plays a critical role in the pathogenesis and progression of NAFLD by regulation of gut permeability, changes in luminal metabolism of bile acid and food substrates, and production of lipoprotein lipase, endogenous alcohol, and toxic compounds [4–9]. Increased gut permeability and lipoprotein lipase production with gut dysbiosis contribute to NAFLD pathogenesis [5]. Gut microbiota involved in bile acid biosynthesis, which influences NAFLD progression by regulating farnesoid *X* receptor (FXR) in the liver, and hepatic steatosis resulting from a high-fat diet (HFD), were reversed upon inhibition of intestinal FXR through alterations in the gut microbiota upon antibiotic administration [4, 5]. The production of short-chain fatty acids (SCFAs) and carbohydrate fermentation by gut microorganisms help inhibit lipid synthesis and accelerate lipid oxidation in the liver [4, 5]. Some enzymes produced by the gut microbiota convert dietary choline into toxic compounds (such as methylamines), which are taken up by the liver and cause liver injury and inflammation [6]. Moreover, the major source of endogenous alcohol is the gut microbiota, and numerous alcohol-producing bacteria have been reported in NASH patients [7, 8]. Alleviation of NAFLD through probiotic or prebiotic treatments is effective in animals and humans, which further confirms the effect of gut microbiota on NAFLD [9]. Hence, modulation of the gut microbiota is a potential method to treat NAFLD.

Glucagon-like peptide-1 receptor agonist (GLP-1R) liraglutide, an antidiabetic agent for T2DM patients, reportedly exerts beneficial effects in NAFLD [10–12], with the underlying mechanisms potentially involving body weight reduction, improved blood glucose regulation, decreased lipid synthesis, autophagy induction, and free fatty acid *β*-oxidation [10, 13]. Dietary intervention, prebiotics, and probiotics reduce body weight and improve metabolic disorders through modulation of gut microbiota [14–16]. Recent studies report that liraglutide is beneficial for weight loss through modulation of the structure of gut microbiota in simple obese and diabetic obese rodents [17–19]. However, it is unknown whether NAFLD attenuation by liraglutide is also associated with modulation of the structure of the gut microbiota. Hence, the present study aimed to investigate the effect of liraglutide on the intestinal microbiota in rats with HFD-induced NAFLD through next-generation sequencing and multivariate analysis.

## 2. Materials and Methods

### 2.1. Animals and Treatments

Male Sprague-Dawley rats (*n* = 24, 4-week-old, Shanghai Jiesijie Laboratory Animal Co. Ltd. Shanghai, China) were housed in a controlled environment (12 h day/night cycle, lights off at 18 : 00 h) with ad libitum access to food and water. After 1 week of acclimatization under laboratory conditions, the rats were randomly segregated into two groups: a normal control group (NC, *n* = 8) administered a standard chow diet comprising 10% fat, 64% carbohydrate, and 26% protein; and an HFD group (HFD, *n* = 16) administered an HFD comprising 45% fat, 37% carbohydrate, and 18% protein. Two rats from each group were euthanized to determine whether NAFLD was successfully established after 12 weeks. The remaining rats of the HFD group were equally assigned to two groups: the HFD group (*n* = 8) or the HFD + liraglutide (H + L) group (*n* = 8). The latter received a daily subcutaneous injection of liraglutide (Novo Nordisk, Copenhagen, Denmark, 0.2 mg/kg body weight). The other two groups (NC and HFD) received saline. Body weight was monitored once a week. Animals were euthanized at 4 weeks after initial liraglutide or saline administration. Blood and gut content samples were harvested and stored at −80°C. The liver and intestine were precisely dissected out, weighed, washed with PBS, and stored at −80°C. This study conformed to the guidelines established by the Research Animal Care Committee of Drum Tower Hospital affiliated with the Medical School of Nanjing University, Nanjing, China.

### 2.2. Histopathological Analysis

Rat livers were carefully dissected out, fixed with formalin solution, and embedded in paraffin. Tissue sections of livers were prepared and stained with hematoxylin and eosin, using a standard protocol. Formalin-fixed tissue sections were then stained with Oil Red O (Sigma, San Francisco, USA) in accordance with the standard protocol [20].

### 2.3. Assessment of Metabolic Profiles and Inflammation Markers

For each rat, commercial ELISA kits were utilized to quantify the level of liver triglycerides (TGs) and serum tumor necrosis factor-*α* (TNF-*α*; Applygen Technologies, Beijing, China), and serum aspartate aminotransferase (AST), alanine (ALT), lipopolysaccharide (LPS), and diamine oxidase (DAO; USCN, Wuhan, China), in accordance with the manufacturer's instructions.

### 2.4. Transmission Electron Microscopy

Tight junction structures were examined via transmission electron microscopy. In brief, ileal specimens were fixed in 4% paraformaldehyde, postfixed in 1% osmium tetroxide, dehydrated using a graded alcohol series, embedded in epoxy resin, stained with uranyl acetate citrate, and examined using a transmission electron microscope, as previously described [19].

### 2.5. DNA Extraction, PCR Amplification, and MiSeq Sequencing

Fecal samples were stored at −80°C until DNA extraction. Total genomic DNA was extracted from each sample using a QIAamp DNA Stool Mini Kit in accordance with the manufacturer's instructions. PCR amplification and MiSeq sequencing were performed as previously described [20]. In brief, the V4–V5 regions of bacterial 16S rDNA were amplified using the Phusion High-Fidelity PCR Master Mix with HF buffer (New England Biolabs, UK). Barcode-indexed PCR primers 515F and 926R were used. Amplicon libraries were purified using the AXYGEN AxyPrep DNA Gel Extraction Kit (Axygen Scientific, Union City, CA, USA), normalized via FTC-3000TM real-time PCR, and sequenced using a MiSeq instrument (Illumina) using a 2 × 300-cycle V3 kit.

### 2.6. Bioinformatic Analysis

The raw sequencing reads were optimized and subjected to bioinformatic analysis as previously described [20]. In brief, the raw data were demultiplexed in accordance with the barcode. Low-quality base pairs were eliminated using Trimmomatic (version 0.35). Trimmed reads were merged and screened using FLASH (version 1.2.11) and Mothur (version 1.33.3), respectively. Multivariate statistical analyses were performed using Mothur, UPARSE (usearch version v8.1.1756), and R (version 3.2.3). Clean tags were clustered into OTUs and then assigned to their corresponding taxa in accordance with the Silva 119 database. Multivariate analyses were performed to evaluate overall structural changes in the gut microbiota, and a rarefaction curve and alpha-diversity were used to assess the richness and diversity of the microbiota in each group, including UniFrac distance-based principal coordinate analysis (PCoA) and a UniFrac tree. The *α*-diversity and *β*-diversity were analyzed using Mothur and R.

### 2.7. Statistical Analyses

Data were analyzed using Student's *t*-test or one-way analysis of variance with Bonferroni post hoc tests, using SPSS Statistics 19. Spearman's correlation analysis was performed to determine correlations between microbial communities and metabolic parameters. Data are expressed as mean ± standard deviation values, and a *P* value (*P* < 0.05) was considered statistically significant.

## 3. Results

### 3.1. Effects of Liraglutide on Hepatic Steatosis, Intestinal Barrier Function, and Inflammatory Levels in High-Fat-Diet Rats

**Figure 1:**
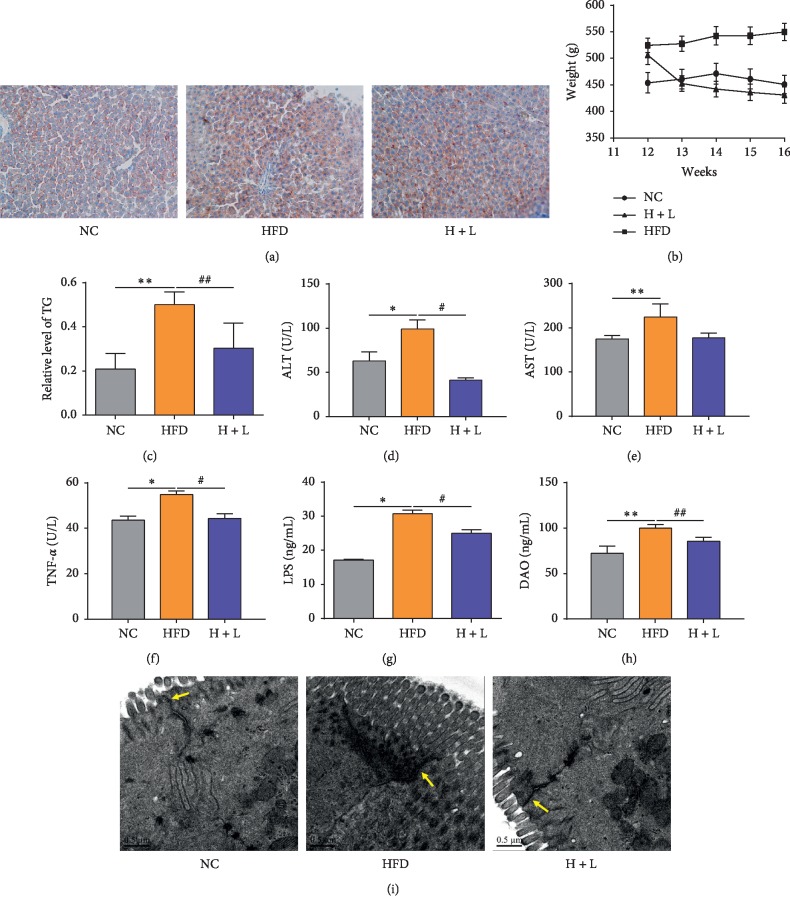
Liraglutide attenuates nonalcoholic fatty liver disease, retains intestinal barrier function, and decreases inflammation levels in rats on a high-fat diet. (a) Oil Red O staining of liver sections; (b) body weight of rats; (c) relative levels of triglycerides (TG) in the liver; (d) levels of serum alanine transaminase (ALT), (e) aspartate transaminase (AST), (f) tumor necrosis factor-*α* (TNF-*α*), (g) lipopolysaccharide (LPS), and (h) diamine oxidase (DAO); (i) ultrastructure of tight junctions in the ileal mucosa (transmission electron microscopy, 20,000x). Data are expressed as mean ± standard deviation values. ^*∗*^*P* < 0.05 vs NC group; ^*∗∗*^*P* < 0.01 vs NC group; ^#^*P* < 0.05 vs HFD group; ^##^*P* < 0.01 vs HFD group.

**Figure 2:**
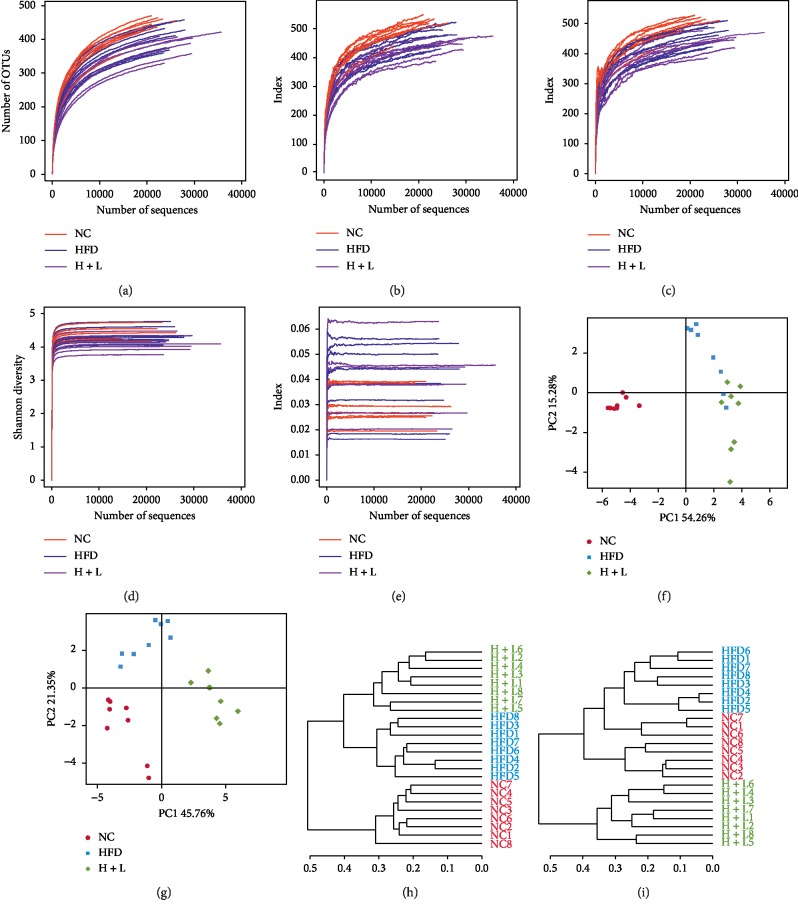
*α*-Diversity analysis and structural changes in the gut microbiota upon liraglutide administration. (a) Rarefaction curves of multiple samples; (b) Chao index curve; (c) Ace index curve; (d) Shannon curve; (e) Simpson index curve; principal coordinate analysis (PCoA) score plot based on unweighted (f) and weighted (g) PCoA scores. UniFrac tree based on unweighted (h) and weighted (i) PCoA scores.

HFD rats displayed a slightly distorted structure of the hepatic lobule and increased deposition of lipid droplets (Figure 1(a)), significantly increased liver TG content (*P* < 0.05; Figure 2(c)), and significantly higher serum ALT (*P* < 0.05; Figure 2(d)) and AST levels (*P* < 0.01; Figure 2(e)) in comparison with control rats. Furthermore, levels of intestinal barrier function, endotoxin, and inflammatory factors in the serum, such as DAO, LPS, and TNF-*α* (*P* < 0.01 − 0.05), were significantly increased in HFD rats in comparison to control rats (Figures 1(f)–1(h)). Transmission electron microscopy revealed that the width of tight junctions in ileal tissue was broadened in HFD rats (Figure 1(i)). However, 4-week liraglutide administration significantly reversed all these changes (*P* < 0.01 − 0.05), except for AST levels (Figures 1(a), 1(c)–1(i)). Additionally, there is a stable decrease in body weight during the treatment of liraglutide (Figure 1(b)).

### 3.2. Liraglutide Altered the Composition of the Gut Microbiota

High-throughput sequencing yielded 593,121 high-quality sequences and 725 OTUs from 24 fecal samples. Rarefaction curves indicated that the current sequencing depth was adequate, and only a few new OTUs were obtained through subsequent sequencing (Figure 2(a)). Chao and ACE analyses revealed that the richness of the gut microbiota was significantly lower in the HFD group than in the control group (HFD vs. NC, *P*=0.039 for the Ace index and *P*=0.039 for the Chao index), while no significant difference was observed between the H + L and HFD groups (Figures 2(b) and 2(c)). Shannon and Simpson's analysis revealed that the overall microbial diversity did not differ significantly among the three groups (Figures 2(d) and 2(e)).

According to the unweighted and weighted PCoA score plot, structures of the gut microbiota were altered along with the second principal component (PC2) in the HFD group relative to the control group, whereas these changes were reversed upon liraglutide administration (Figures 2(f) and 2(g)). The unweighted and weighted UniFrac trees revealed that there are three different communities of microbiota among the groups (Figures 2(h) and 2(i)).

Taxon-based analysis was performed to further explore changes among the three groups. These OTUs comprised 13 phyla. The major phyla were Firmicutes, Bacteroidetes, Actinobacteria, Spirochaetes, and Proteobacteria. Taxon-based analysis revealed that liraglutide significantly altered the composition of the gut microbiota in HFD rats. Consequently, bacteria of phylum Bacteroidetes (*P*=0.002), Tenericutes (*P*=0.002), Cyanobacteria (*P*=0.002), Elusimicrobia (*P*=0.03), and Fusobacteria (*P*=0.002) were significantly decreased, while those of phylum Firmicutes (*P*=0.002), Actinobacteria (*P*=0.003), Proteobacteria (*P*=0.018), and Deinococcus-Thermus (*P*=0.049) were significantly increased in the H + L group relative to the HFD group.

**Figure 3:**
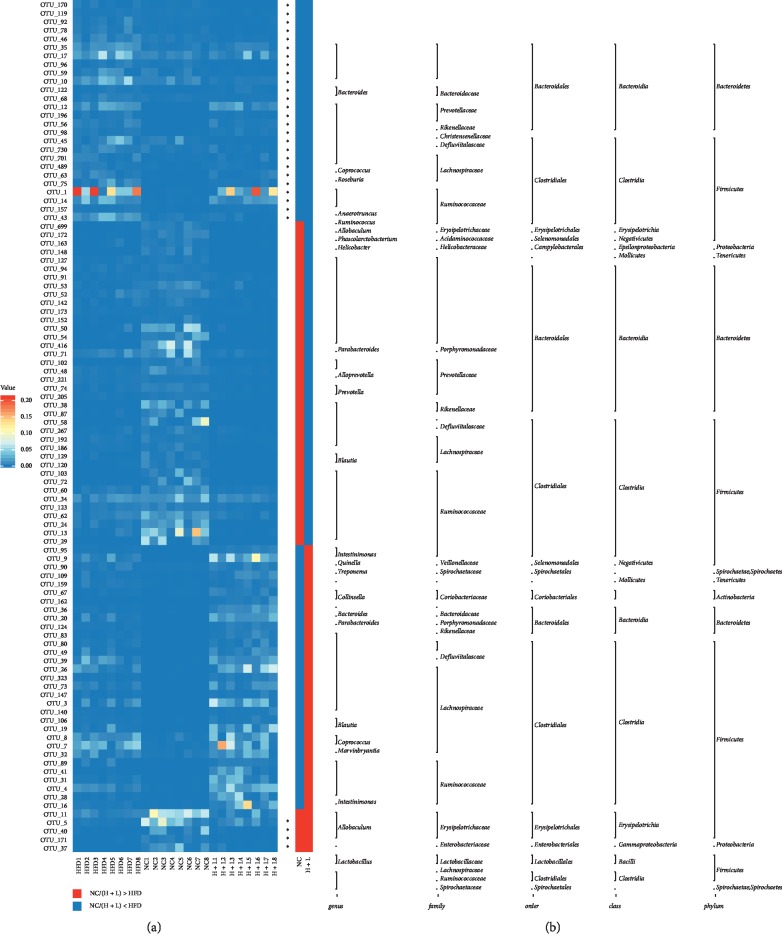
An abundance of 100 operational taxonomic units (OTUs) was altered upon liraglutide administration. Indicated are the OTUs that were more (red) and less abundant (blue) in the high-fat diet (HFD) + liraglutide group and the control group relative to the HFD group, respectively. (a) Heatmap of 100 OTUs. (b) Altered direction of the 100 OTUs upon liraglutide administration. Taxa of the OTUs (genus, family, and phylum) are shown on the right side. The asterisk (^*∗*^) represents OTUs wherein abundance was altered via the HFD and reversed upon liraglutide administration.

Hundred OTUs of gut microbiota were significantly altered, among which 57 OTUs were increased and 43 OTUs were decreased in the HFD group in comparison with the control group (Figure 3). Twenty-six reduced OTUs in the HFD group were increased upon liraglutide administration, while 5 increased OTUs were decreased. Among the remaining 69 OTUs, 38 were increased and 31 were decreased upon liraglutide administration. Among the 38 increased OTUs, 4, 6, and 11 OTUs were classified into family Erysipelotrichaceae, Ruminococcaceae, and Lachnospiraceae, respectively. Among the 31 decreased OTUs, 11, 4, and 5 OTUs were classified into families Ruminococcaceae, Lachnospiraceae, and Prevotellaceae, respectively. Hence, we speculated that the genus and species potentially influence NAFLD. Among the 38 increased OTUs, 6 putative SCFA-producing bacteria, including those of genera *Allobaculum* (OTU_4, OTU_31, and OTU_28) and *Bacteroides* (OTU_109) and species *blautia* (OTU_58, OTU_87), were markedly increased upon liraglutide administration (Figure 3). Furthermore, we speculated that SCFA-producing bacteria constitute an important factor contributing to the beneficial effects of liraglutide. Together, these results indicate that liraglutide modulates the predominance of OTUs in a strain-specific manner, resulting in a microbiota composition similar to that of control rats.

### 3.3. Associations between the 78 OTUs and Metabolic Parameters Were Altered upon Liraglutide Administration

**Figure 4:**
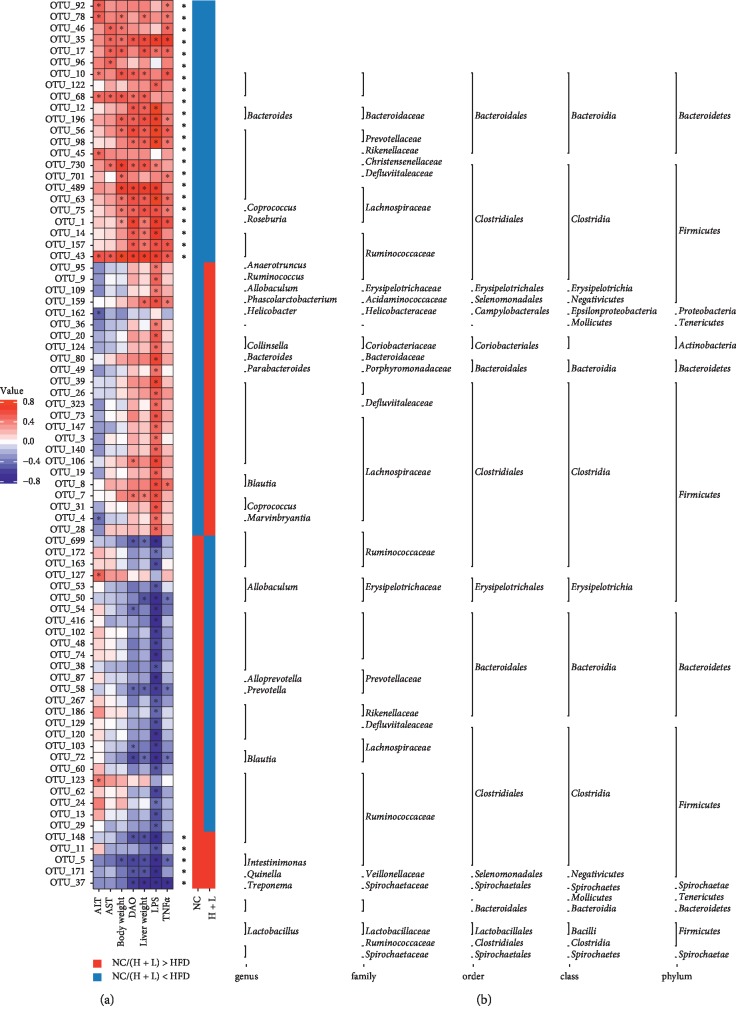
Seventy-eight operational taxonomic units (OTUs) altered upon liraglutide administration were significantly correlated with host metabolic parameters in accordance with Spearman's correlation analysis. (a) Correlation between 76 OTUs and host metabolic parameters. Rows correspond to OTUs with identities shown on the left and columns correspond to metabolic parameters. Red and blue colors indicate positive and negative associations, respectively. Color intensity represents the degree of association between the OTU abundances and host parameters, as assessed via Spearman's correlation analysis. Asterisks indicate significant associations. Taxonomic classification of the OTUs is shown on the right side. (b) Altered direction of 76 OTUs. Red and blue colors indicate the OTUs that were more and less abundant, respectively, in the H + L and control groups in comparison with the high-fat diet (HFD) group. The asterisk (^*∗*^) represents OTUs whose level was altered via an HFD and then reversed significantly upon liraglutide administration.

Seventy-eight OTUs were significantly correlated with at least one abnormal metabolic parameter, including ALT, AST, DAO, body weight, liver weight, LPS, and TNF-*α* (Figure 3). Twenty-three OTUs decreased in response to an HFD were increased upon liraglutide administration (Figure 4), of which two were positively correlated with at least one abnormal metabolic parameter. Twenty-one of the 23 reduced OTUs were positively correlated with at least two abnormal metabolic parameters. Five OTUs increased with an HFD were decreased upon liraglutide administration, one of which was negatively associated with at least one abnormal metabolic parameter, and the remaining 4 were negatively correlated with at least two abnormal metabolic parameters.

## 4. Discussion

This study shows that liraglutide alleviates NAFLD and is beneficial for weight loss, maintenance of intestinal barrier function, and reduction of inflammation levels via modulation of the structure of gut microbiota in obese rats on an HFD. Gut microbiota associated with metabolic parameters contributed to liraglutide-mediated attenuation of NAFLD, consistent with prior reports that changes in gut microbiota composition and activity are associated with metabolic disorders, such as obesity, diabetes, and cardiometabolic disorders.

Liraglutide potentially helps attenuate NAFLD; however, its underlying mechanism is yet unclear [21]. Weight loss is currently known as the only effective strategy to improve NAFLD, and in the present study, liraglutide treatment resulted in significant weight loss [13, 22, 23]. Hence, weight loss potentially contributes to liraglutide-mediated attenuation of NAFLD. Consistent with previous reports, this study shows that liraglutide induces weight loss and attenuates NAFLD. However, the mechanism underlying weight loss resulting from liraglutide administration is unclear. The side effects of liraglutide potentially include nausea and vomiting, thus resulting in weight loss. Lean et al. reported an average weight loss of 9.2 kg vs 6.3 kg in patients with or without nausea/vomiting episodes after one year of liraglutide treatment [24]. However, the 6.3-kg weight loss in patients without nausea/vomiting after liraglutide treatment lacked a reasonable explanation.

Other studies have focused on the activating effects of the GLP-1 receptor on hepatocytes [25]. However, it is still controversial whether GLP-1 receptors are present on human hepatocytes [26, 27]. Recent studies have reported that GLP-1 prevents diabetes by altering the structure of the gut microbiota and inhibiting inflammation [28, 29]. Inflammation, caused by a high-fat diet, is one factor inducing metabolic disorders [30]. Previous studies reported that LPS/TLR4 signaling contributes significantly to NAFLD pathogenesis [31, 32]. DAO, a marker for the assessment of gut barrier function, enters the bloodstream when the gut barrier is impaired [33]. LPS crosses the gut barrier and penetrates the liver from the portal circulation. Activated LPS/TLR4 signaling in Kupffer cell causes insulin resistance through inhibition of insulin receptor substrate-1 phosphorylation in the liver [34–36]. However, it is still unknown whether liraglutide attenuates NAFLD through reduction of inflammation. Our results show that serum levels of metabolic endotoxemia and inflammation markers, including LPS, TNF*α*, and DAO, increased in HFD rats relative to control rats upon liraglutide administration, indicating that liraglutide attenuates NAFLD by potentially accelerating weight loss and inhibiting low-grade chronic inflammation. However, the question regarding how these alterations are brought about remains.

To determine the reasons for the weight loss and inhibition of inflammation, the gut microbiota of the rats was analyzed. As revealed through the analysis of α-diversity, microbial enrichment was markedly decreased in NAFLD rats and was not reversed upon liraglutide administration. However, *β*-diversity analysis revealed that the composition of the gut microbiota was markedly altered upon liraglutide administration. Hence, we speculated that a “more healthy composition of the gut microbiota,” which benefited lipid metabolism and inhibition of inflammation, was potentially acquired upon liraglutide administration, rather than recovery of the original composition similar to that in control rats.

Taxon-based analysis revealed that at the phylum level, liraglutide administration significantly increased bacteria of phylum Firmicutes and Actinobacteria and significantly decreased those of phylum Bacteroidetes and Tenericutes. Increased Firmicutes/Bacteroidetes ratio results in decreased SCFA production and increased energy harvested from the diet, facilitating the development and progression of NAFLD [5]. Some studies have reported that a higher Firmicutes/Bacteroidetes ratio was decreased by liraglutide in obese and diabetic rats [17, 28, 29]. However, the relationship of Firmicutes/Bacteroidetes ratio with obesity and NAFLD remains controversial [37–39]. These results indicate that further classification may yield exact reasons. *Lactobacillus*, a genus of Gram-positive, nonsporulating, anaerobic bacillus, generally used as probiotics, efficiently improves NAFLD [6]. In the present study, liraglutide administration significantly increased bacteria of genus *Lactobacillus* in HFD rats. Another study confirmed that liraglutide helped decrease the genus *Helicobacter* and increase *Akkermansia muciniphila* in the HFD group [37]. Different OTUs from the same genus displayed different responses to liraglutide treatment, indicating that liraglutide differently regulated the gut microbiota by targeting different species even from the same genus. Therefore, Spearman's correlation analysis was performed to determine which OTU was important in causing obesity and inflammation. Consistent with another study [37], the analysis revealed that liraglutide altered microbial communities and these change were related to weight loss and reduced inflammation levels. Five OTUs belonging to the genus *Lactobacillus*, family Ruminococcaceae, family Spirochaetaceae, and order Bacteroidales exerted beneficial effects in NAFLD. Furthermore, 23 OTUs belonging to the genus *Bacteroides*, *Coprococcus*, *Roseburia*, *Anaerotruncus*, and *Ruminococcus* exerted negative effects in NAFLD. The effect of the GLP-1R agonist on the gut microbiota structure might relate to the reduction of food intake and gastrointestinal motility, and the changes in diet composition [37].

## 5. Conclusions

In conclusion, liraglutide attenuates NAFLD by decreasing body weight and inhibiting inflammation through alterations in the gut microbiota. Further studies are required to explore the specific mechanism by which liraglutide affects the intestinal microbiota in humanized mice and obese humans.

## Data Availability

The data used to support the findings of this study are available from the corresponding author upon request.

## Abbreviations

ALT: Alanine transaminase
AST: Aspartate transaminase
DAO: Diamine oxidase
FXR: Farnesoid X receptor
HFD: High-fat diet
LPS: Lipopolysaccharide
NAFLD: Nonalcoholic fatty liver disease
NASH: Nonalcoholic steatohepatitis
OUT: Operational taxonomic unit
PCoA: Principal coordinate analysis
SCFA: Short-chain fatty acid
T2DM: Type 2 diabetes mellitus
TG: Triglyceride
TNF: Tumor necrosis factor.
